# Layer‐specific effects of dopaminergic D1 receptor activation on excitatory synaptic trains in layer V mouse prefrontal cortical pyramidal cells

**DOI:** 10.14814/phy2.13806

**Published:** 2018-08-02

**Authors:** Jonna M. Leyrer‐Jackson, Mark P. Thomas

**Affiliations:** ^1^ University of Northern Colorado School of Biological Sciences University of Northern Colorado Greeley Colorado

**Keywords:** AMPA, EPSP, NMDA, postsynaptic potentials

## Abstract

In humans, executive functions (e.g., working memory [WM]) are mediated in part by prefrontal cortical areas (PFC), where ventromedial areas may be homologous to ventromedial areas (mPFC) in rodents. Many executive functions are critically dependent on optimal dopamine levels within the PFC; however, our understanding of the role of dopamine in modulating PFC‐mediated tasks is incomplete. Stable patterns of neuronal activity have been associated with WM processes, and recurrent excitatory synaptic activity has been proposed to play a role in this sustained activity. This excitatory activity may be regulated in a frequency‐dependent manner. Thus, we examined the effects of dopamine D1‐like receptor (D1R) activation on short‐term excitatory postsynaptic potential (EPSP) dynamics in two subtypes of mouse layer V mPFC pyramidal neurons by varying evoked train frequency from 10 to 50 Hz. We isolated non‐NMDA receptor (non‐NMDAR) and NMDA receptor (NMDAR)‐mediated components of EPSP trains, which were evoked by stimulating fibers located either within layer V or layer I of the mPFC. Interestingly, no differences in the effects of D1R activation were observed between subcortically projecting (PT or pyramidal tract) and contralaterally projecting (IT or intratelencephalic) layer V pyramidal cells. However, we found that D1R activation had layer‐specific effects on NMDAR‐ and non‐NMDAR‐mediated EPSP trains: while D1R activation increased the amplitude of both components with layer V stimulation, with layer I stimulation D1R activation had no effect on non‐NMDAR‐mediated EPSP trains but decreased the amplitude of NMDAR‐mediated EPSP trains. Our results suggest that dopamine, acting at D1‐like receptors, increases the influence of local inputs from other layer V pyramidal cells, but may restrict the influence of layer I (tuft) inputs. Our demonstration of differential D1R regulation of excitatory synaptic dynamics in distinct compartments of mPFC layer V neurons may provide another important aspect linking cellular mechanisms of dopaminergic modulation to PFC network functioning, and ultimately to executive functions such as working memory.

## Introduction

In humans, executive functions are mediated by prefrontal cortical (PFC) regions, in concert with posterior cortical regions. The goal‐directed behaviors mediated by PFC regions allow for flexible behavior in the face of constantly changing environmental demands. The ventromedial regions of the PFC are thought to mediate action‐outcome associations (Euston et al. 2012). These functions are mediated by the medial prefrontal cortex (mPFC) in rodents, hypothesized to be homologous with specific human and primate ventromedial regions (Heidbreder and Groenewegen 2003; Seamans et al. 2008). It is known that optimal levels of dopamine, released within the PFC from afferents originating in the ventral tegmental area, are essential for normal executive tasks (Goldman‐Rakic 1995). Acting through two major subtypes of receptors (D1‐like and D2‐like), dopamine plays roles in prefrontal functions such as updating working memory (Sawaguchi and Goldman‐Rakic 1991), rewarding appetitive behaviors (Sawaguchi and Goldman‐Rakic 1991) and updating contextual representations (D'Ardenne et al. 2012). Dysregulation of dopaminergic inputs to the PFC contributes to deficits in working memory and social withdrawal, symptoms commonly observed in patients diagnosed along the schizoaffective‐schizophrenic spectrum of disorders, as well as the mood disturbances and cognitive symptoms observed in bipolar disorder.

Layer V prefrontal cortical cells, the major neocortical output cells, are comprised of at least two subtypes: subcortically projecting (type I or PT, pyramidal tract) and contralaterally projecting (type II or IT, intratelencephalic) (Molnár and Cheung 2006; Dembrow et al. 2010; Lee et al. 2014). These cells are known to differ in hodology, dendritic morphology, and intrinsic properties. Additionally, Gee et al. (2012) provided evidence that type I and type II cells differ in their expression of dopamine receptors, where type I cells express both D1‐like and D2‐like receptors, and type II cells express only D1‐like receptors. Given that both D1‐like and D2‐like receptors play a role in WM functions, dopamine may have differential effects on layer V pyramidal subtypes that relate to their different roles in WM functions.

Layer V neocortical pyramidal neurons receive compartmentalized inputs from various brain regions, which provide feed‐back (top‐down or contextual) information to the apical tuft region (within layer I), and feed‐forward (bottom‐up or current environmental) information to the basal dendrites, a compartment where local processing between layer V cells is also prominent (e.g., Larkum 2013). When these signals coincide (i.e., synaptic inputs to the apical tufts are activated nearly synchronously with postsynaptic action potentials), pyramidal cells act as coincidence detectors, firing several spikes at high frequency (Larkum et al. 2009). It has been hypothesized that this pattern of activity may play a role in the main function of cortex, which is to “associate external data with an internal representation of the world” (Larkum et al. 2009). It seems likely that this high‐frequency activity could also promote synaptic plasticity, locally and in target neurons. Additionally, a subset of layer V pyramidal cells within the PFC can fire persistently during the delay phase of a working memory task (Goldman‐Rakic 1995). In this study, we aimed to explore phenomena relating to the hypothesis that frequency‐dependent, layer‐specific, short‐term synaptic dynamics and their modulation by D1 receptor activation play a significant role in generating the neural activity observed in prefrontal cortical networks during memory tasks (Goldman‐Rakic 1995).

Excitatory postsynaptic potentials (EPSPs) are characterized by two components, a non‐NMDA receptor (non‐NMDAR)‐mediated (primarily AMPA receptor) and an NMDA receptor (NMDAR)‐mediated component that confer fast synaptic transmission and synaptic plasticity, respectively. In a companion paper, we have characterized the effects of D2 receptor activation on short‐term synaptic dynamics in layer V pyramidal cells (Leyrer‐Jackson and Thomas 2017). Studies characterizing dopaminergic D1 receptor modulation of excitatory transmission have primarily focused on synaptic responses evoked at low frequencies and have yielded inconsistent results (Gao et al. 2001; Young and Yang 2005; Matsuda et al. 2006). In this study, we aimed to determine the effects of D1 receptor activation on isolated non‐NMDAR‐ and NMDAR‐mediated responses over a range of frequencies that mimic high‐frequency activity, in both layer V pyramidal cell subtypes. We also examined the effects of D1 receptor activation on layer I and layer V evoked EPSP trains, reflecting feed‐back and feed‐forward information processing, respectively. The intent of this study was to describe the overall effects of D1 receptor activation on synaptic dynamics in the two major dendritic compartments of mPFC layer V pyramids, without addressing subcellular mechanisms of D1 receptor modulation. Some of these results have been presented previously in abstract form (Leyrer‐Jackson and Thomas 2014).

## Materials and Methods

### Tissue preparation

Tissue slices were prepared from 25‐ to 42‐day‐old male and female mice (C57 BL/6 strain, UNC breeding colony). Animals were anesthetized with carbon dioxide and rapidly decapitated following procedures outlined in a UNC Institutional Animal Care and Use Committee approved protocol in accordance with NIH guidelines. Brains were rapidly removed and immersed in ice‐cold carbogen (95% O_2_/5% CO_2_) saturated sucrose‐enriched artificial cerebrospinal fluid (cutting aCSF) containing (in mmol/L): sucrose, 206; NaHCO_3_, 25; dextrose, 10; KCl, 3.3; NaH_2_PO_4_, 1.23; CaCl_2_, 1.0; MgCl_2_, 4.0, osmolarity adjusted to 295 ± 5 mOsm and pH adjusted to 7.40 ± 0.03. The brains were then transferred to the cutting chamber of a vibrating tissue slicer (OTS500, Electron Microscopy Sciences, Hatfield, PA) and coronal slices of the prefrontal cortex (PFC) were prepared in ice‐cold cutting aCSF. Slices were cut 300 *μ*m thick and were taken from approximately 200 *μ*m to 1400 *μ*m caudal to the frontal pole. Slices were then placed in a holding chamber filled with recording aCSF solution containing (in mmol/L): NaCl, 120; NaHCO_3_, 25; KCl, 3.3; NaH_2_PO_4_, 1.23; CaCl_2_, 0.9; MgCl_2_, 2.0; dextrose, 10, osmolarity adjusted to 295 ± 5 mOsm and pH adjusted to 7.40 ± 0.03. The holding chamber aCSF was continuously bubbled with carbogen and incubated at 34°C for 45 min and then allowed to cool to room temperature before slice recording. Prior to experiments, slices were transferred to a recording chamber where they were perfused continuously at a flow rate of 1–2 mls/min with filtered, carbogen‐saturated recording aCSF solution.

Throughout recordings, the recording chamber was held at 32 ± 1°C with a temperature controller equipped with a chamber heater and an in‐line heater (TC‐344B, Warner Instruments, Hamden CT). In experiments isolating non‐NMDAR‐mediated EPSPs, the recording aCSF contained 50 *μ*mol/L aminophosphonovalerate (D‐APV;an NMDA receptor antagonist). In experiments isolating NMDAR‐mediated EPSPs, the recording aCSF contained 20 *μ*mol/L 6,7dinitroquinoxaline‐2,3‐dione (DNQX; a non‐NMDA receptor antagonist) and MgCl_2_ concentration was reduced to 0.25 mmol/L to facilitate NMDAR activation at −65 mV.

### Electrophysiology

**Figure 1:**
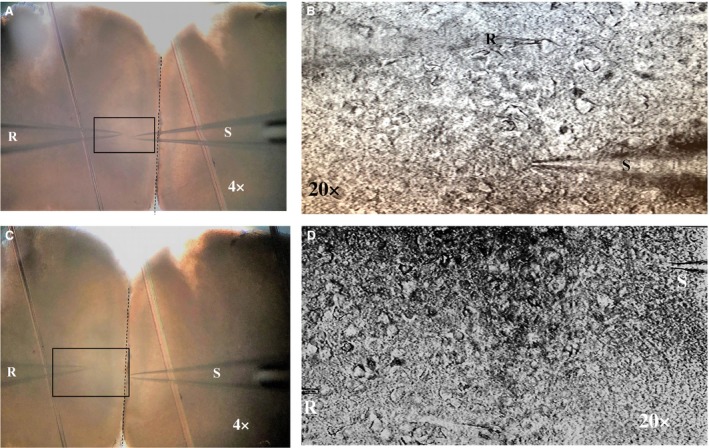
Recording and stimulating electrode placement. Recordings were made from the soma of both type I and type II layer V pyramidal cells with a recording electrode (R). The stimulating electrode (S) was placed within layer V (A and B) or layer I (C & D), approximately 50–100 *μ*m from the recorded cell. Magnifications of 4X and 20X are shown for both layer V and layer I stimulations.

Layer V pyramidal neurons of the infralimbic, prelimbic, and anterior cingulate cortices were visually identified using infrared DIC microscopy at 400x magnification with an Olympus BX51WI microscope (Tokyo, Japan). Whole‐cell recordings were made from the soma of layer V pyramidal neurons after establishing a Giga‐ohm seal (resistance range: 1–10 Gohm). Only cells that exhibited a thin (i.e., action potential half‐width was <2 msec), overshooting action potential, as well as continuous spiking throughout a depolarizing current injection were used in this study. Access resistance (*R*_A_) was compensated throughout experiments, and cells were excluded from analysis if uncompensated R_A_ exceeded 20 MΩ. Liquid junction potentials (estimated at approximately −6 mV for K^+^ gluconate internal solution) were not compensated in adjusting *V*m for synaptic recordings. Amplifier bridge balance was utilized and monitored throughout current injections. Recording pipettes (4–6 MΩ tip resistance), produced from thin‐wall glass capillary tubes (1.5 *μ*m OD, 1.12 *μ*m ID, World Precision Instruments, Sarasota, FL), were filled with (in mmol/L) potassium gluconate, 135; KCl, 10; EGTA, 1.0; HEPES, 10; MgATP, 2; TrisGTP, 0.38, osmolarity adjusted to 285 ± 5 mOsm and pH adjusted to 7.30 ± 0.01. Glass micropipettes, used as stimulating electrodes, were filled with 3 mol/L NaCl and placed within either layer V or layer I of mPFC to activate fibers located within that layer (Fig. 1).

Type I and type II layer V pyramidal cells were identified based on the presence of a prominent “sag” in response to a 150 pA hyperpolarizing current (type I: minimal 12% depolarization from peak of hyperpolarization, indicating the strong presence of the hyperpolarization activated cation current), and by initial firing of doublets (type I cells only); both criteria have been used in previous studies (Dembrow et al. 2010; Spindle and Thomas 2014). For all analyses, type I and type II subtypes were categorized and compared between experimental groups. The responses were digitized at 10 kHz and saved on disk using a Digidata 1322A interface (Axon instruments) and pCLAMP version 8.1 software (Clampex program, Axon Instruments). Data were analyzed off‐line in Clampfit (Axon Instruments).

### Statistical analyses

All values are presented as mean ± SEM (standard error of the mean). All cells received every stimulus frequency (10–50 Hz) and the D1R agonist. We performed an ANOVA on all experimental data except for comparisons between cellular properties of type I and type II cells (i.e., resting membrane potential, membrane capacitance, ‘sag’ amplitude etc.), where a Student's *t*‐test was utilized. A two‐way repeated measures ANOVA was used to analyze the effects of drug and frequency of stimulation. The statistical model also included mouse and slice as random variables.

### Experimental protocols

We began each experiment by establishing a “stimulus current / evoked response” curve where stimulus intensity was increased while measuring the evoked EPSP amplitude. The stimulus current was adjusted to establish an unsaturated response near the midrange of this curve. The position of the stimulating pipette was located at a distance from the recorded cell to establish a baseline response of approximately 7–13 mV and 2–7 mV (for non‐NMDAR‐ and NMDAR‐mediated EPSPs, respectively). These amplitudes were chosen to avoid cell spiking during the pulse trains, where summation was often observed at higher stimulus train frequencies. EPSPs were evoked in current clamp mode using an 8‐pulse stimulus train, at varying frequencies (10–50 Hz). This protocol was repeated 5 times with a 10 second inter‐train interval, and the 5 responses were averaged. For non‐NMDAR‐mediated EPSP experiments, cells were manually held at −80 mV throughout the experiment. Pulse trains were applied in control (APV‐containing) aCSF, and again immediately following a 5 min application of the D1 agonist, SKF‐38393 (10 *μ*mol/L). NMDAR‐mediated EPSPs were evoked using the same current clamp protocol as for non‐NMDAR‐mediated EPSP experiments. During NMDAR EPSP experiments, cells were manually held at −65 mV throughout the experiment. Pulse trains were applied in control (DNQX‐containing) aCSF, and again immediately following a 5 min application of the D1 agonist SKF38393 (10 *μ*mol/L). For antagonist experiments, SCH23390 (10 *μ*mol/L) was included in the bath during control recordings.

For analysis, the EPSP amplitude was measure in millivolts (mV), from the membrane potential directly before the stimulus was applied. For the first EPSP within the train, the EPSP size was measured from resting baseline, where simultaneous EPSPs were measured from the baseline directly before the EPSP was evoked. Additionally, maximal peak amplitude was measured from baseline (before the train was delivered), to the peak amplitude reached throughout the train of 8 EPSPs. The time it took to reach the maximal amplitude within the train was also recorded.

### Drugs

The NMDA antagonist, D‐APV, the dopamine D1/D5 receptor agonist, SKF38393, and the dopamine D1/D5 antagonist, SCH23390, were purchased from Tocris Biosciences (Bristol, UK). The non‐NMDA antagonist, DNQX, was purchased from Alomone Labs (Jerusalem, Israel). D‐APV, SKF38393, SCH23390, and DNQX were diluted into aliquots of 50 mmol/L, 10 mmol/L, 10 mmol/L and 20 mmol/L stocks, respectively. All drugs were stored at −80°C and diluted to working concentrations of 50 *μ*mol/L D‐APV, 10 *μ*mol/L SKF38393, 10 *μ*mol/L SCH23390, and 20 *μ*mol/L DNQX; any drugs not used within 3 days of thawing were discarded.

## Results

### Electrophysiological properties differ between type I and type II layer V pyramidal cells

**Figure 2:**
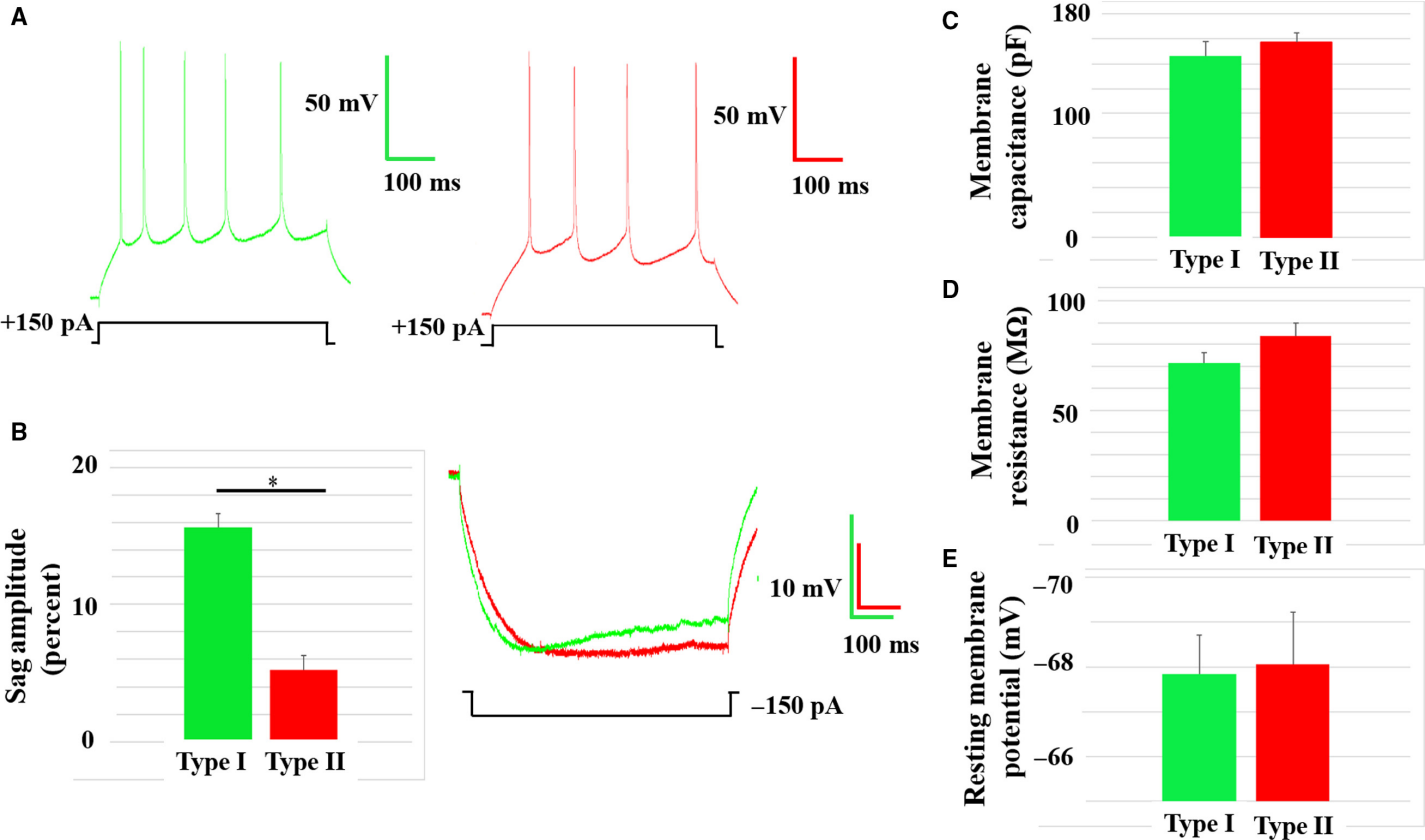
Characteristics of type I and type II pyramidal cells. (A) Type I cell spiking pattern showing a distinct initial spiking doublet and an adapting response to a 150 pA current injection (green; top); Type II cell spiking pattern, lacking an initial spiking doublet with 150pA current injection (red; bottom). (B) Type I and type II cells display significantly different “sag” amplitude in response to a 150 pA hyperpolarizing current (type I: green; type II: red; *P* < 0.05). Representative “sag” traces of type I (green trace) and type II (red trace) cells are shown. Type I cells display a lower, yet non‐significant, average membrane capacitance (type I:146.4 ± 11.4pF; type II: 157.3 ± 7.5pF) (C) and average membrane resistance (mΩ) (type I: 71.5 ± 4.7 mΩ; type II:83.6 ± 6.4 mΩ) (D). (E) Type I cells and type II cells show no difference in membrane potential (type I: ‐67.8 ± 0.9 mV; type II: −68.1 ± 1.2 mV). Asterisks indicate a *P *< 0.05.

As demonstrated in previous studies (Dembrow et al. 2010; Gee et al. 2012; Spindle and Thomas 2014; Leyrer‐Jackson and Thomas 2017), we have also identified two subtypes of layer V pyramidal cells that differ in electrophysiological properties. These subtypes are defined by their axonal projection patterns, where type I cells and type II cells send their axonal projections to the pontine of the brainstem and the contralateral mPFC, respectively (Dembrow et al. 2010; Gee et al. 2012). We have demonstrated that the two cell types differ in spiking characteristics, where type I cells generally display an initial spiking doublet and type II cells do not (Fig. 2A). Additionally, type I cells display a significantly greater “sag” amplitude during a hyperpolarizing stimulus of 150 pA, compared with type II cells (type I: 15.6 ± 1.0%; type II: 5.2 ± 1.0%; *P* < 0.05; Fig. 2B). Although the two cell types differed in some characteristics, they did not differ in membrane capacitance (pF), membrane resistance (MΩ) or resting membrane potential (mV) (Fig. 2C–E, respectively).

### Non‐NMDA‐receptor‐mediated EPSPs

**Figure 3:**
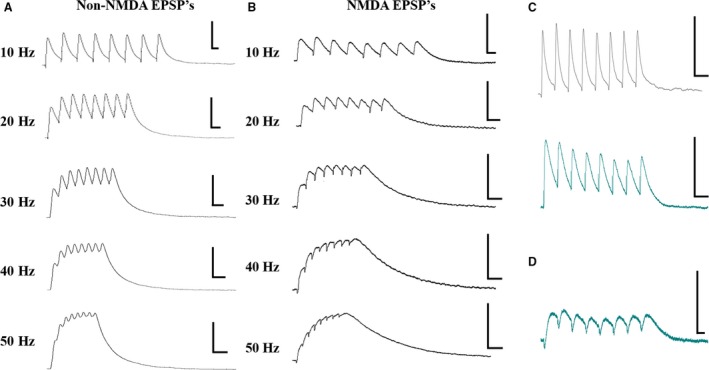
EPSP trains evoked by layer V stimulation. (A) Representative traces showing non‐NMDAR‐mediated EPSP trains evoked by layer V stimulation. Traces are shown for each stimulation frequency (10–50 Hz from top to bottom). (B) Representative traces showing NMDAR‐mediated EPSP trains evoked by layer V stimulation. Traces are shown for each stimulation frequency (10–50 Hz from top to bottom). (C) non‐NMDAR‐mediated EPSP trains show two types of short‐term dynamics, facilitating (EPSP2 > EPSP1; black trace, top) and depressing (EPSP2 < EPSP1; teal trace, bottom). (D) NMDAR‐mediated EPSP trains only displayed depressing (EPSP2 < EPSP1; teal trace, bottom) short‐term dynamics.

Non‐NMDA receptor‐mediated EPSP trains were measured from layer V pyramidal neurons by blocking NMDA receptor activation with 50 *μ*mol/L APV. Representative traces recorded following layer V stimulation are shown for all frequencies (10–50 Hz) in Figure 3A. At 10 Hz, non‐NMDA EPSPs show two distinct amplitude profiles, which we define simply as facilitating (the second EPSP is larger than the first EPSP; EPSP2 > EPSP1) (Fig. 3C, top; black trace) or depressing (the second and subsequent EPSPs are smaller than the first EPSP; EPSP2 < EPSP1) (Fig. 3C, bottom; teal trace). In 12 out of 20 layer V cells (6 type I, 6 type II), layer V‐evoked non‐NMDA EPSPs showed a depressing pattern in control solution. In the remaining 8 of 20 cells (3 type I, 5 type II), layer V‐evoked non‐NMDA EPSPs were facilitating. In 14 out of 16 cells (8 type I, 6 type II), layer I‐evoked non‐NMDA EPSPs were depressing, while only 2 of 16 cells (both type I cells) were facilitating. Notably, we did not see a significant difference between type I and type II cells (as identified by criteria listed in methods) regarding their short‐term synaptic dynamics (layer V evoked non‐NMDA EPSPs [EPSP2/EPSP1; *P* > 0.05; *n* = 20]; layer I evoked non‐NMDA EPSPs [EPSP2/EPSP1; *P* > 0.05; *n* = 16]).

### NMDA‐receptor‐mediated excitatory postsynaptic potentials

NMDA receptor‐mediated EPSP trains were measured from layer V pyramidal neurons by blocking non‐NMDA receptors with 20 *μ*mol/L DNQX. Representative traces recorded for all frequencies (10–50 Hz) following layer V stimulation are shown in Figure 3B. NMDA EPSPs showed only a depressing dynamic profile (EPSP2 < EPSP1; Fig. 3D; teal trace). All NMDA EPSP responses measured in control solution showed depressing profiles, regardless of which layer was stimulated [layer V (*n* = 11; 5 type I, 6 type II); layer I [*n* = 17; 8 type I, 9 type II)]. Again, we did not see a significant difference between type I and type II cells with regard to their short‐term synaptic dynamics (layer V evoked NMDA EPSPs [EPSP2/EPSP1; *P* > 0.05; *n* = 11]; layer I evoked NMDA EPSPs [EPSP2/EPSP1; *P* > 0.05; *n* = 17]).

### Frequency‐dependent properties of EPSP trains

**Figure 4:**
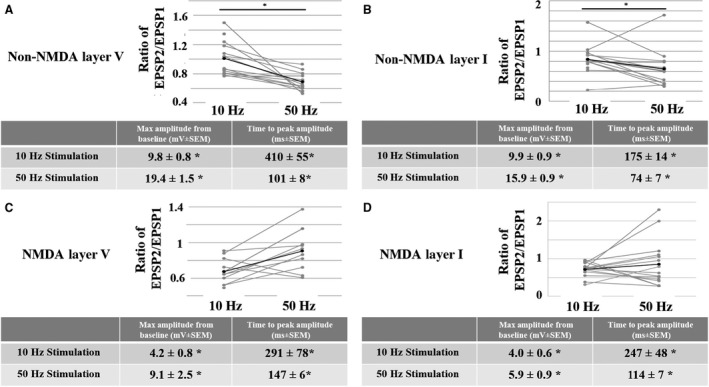
Frequency‐dependent properties of non‐NMDA and NMDA EPSPs evoked by either layer V or layer I stimulation. With layer V (A), but not layer I (B) stimulation, the ratio of EPSP2/EPSP1 is significantly less at 50 Hz compared to 10 Hz for non‐NMDA EPSPs. Additional characteristics are tabled (bottom). With layer V (C), but not layer I (D) stimulation, the ratio of EPSP2/EPSP1 is significantly greater at 50 Hz compared to 10 Hz for NMDA EPSPs. Additional characteristics are tabled (bottom). For each graph, gray lines represent each individual cell and black lines represent the average of all cells. Asterisks in tables and graphs depict a significant difference (*P* < 0.05; RM ANOVA) between 10 Hz and 50 Hz.

We examined the frequency‐dependent short‐term synaptic dynamics of both non‐NMDAR‐ and NMDAR‐mediated EPSPs by stimulating at various frequencies between 10 Hz and 50 Hz. Non‐NMDAR‐mediated EPSPs evoked by layer V and layer I stimulation display a EPSP2/EPSP1 ratio that is significantly less at 50 Hz compared to 10 Hz (Fig. 4A and B, top). Furthermore, for non‐NMDAR‐mediated EPSPs evoked with both layer V and layer I stimulation, maximal amplitude reached during the train was higher, and the latency to peak was shorter, at 50 Hz compared to 10 Hz (Fig. 4A and B, Tables 1, 2, 3, 4, 5). Thus, at higher frequencies, temporal summation of non‐NMDAR EPSPs is more robust.

**Table 1:** D1 receptor activation has frequency‐dependent effects on layer V evoked non‐NMDA EPSPs

	10 Hz stimulation		50 Hz stimulation	
	Max amplitude from baseline (mV ± SEM)	Time to peak amplitude (msec ± SEM)	Max amplitude from baseline (mV ± SEM)	Time to peak amplitude (msec ± SEM)
Control	9.8 ± 0.8^a^	410 ± 55^a^	19.4 ± 1.5	101 ± 8
D1 agonist	15.2 ± 1.7^a^	275 ± 45^a^	20.7 ± 1.7	86 ± 9

The effects of the D1 agonist on maximal amplitude and time to peak amplitude at 10 Hz and 50 Hz are tabled.

a Significant difference between control and D1 receptor activation (*P* < 0.05; RM ANOVA).

**Table 2:** D1 receptor activation enhances max amplitude reached at all frequencies in layer V evoked NMDA EPSPs

	10 Hz Stimulation		50 Hz Stimulation	
	Max amplitude from baseline (mV ± SEM)	Time to peak amplitude (msec ± SEM)	Max Amplitude from baseline (mV ± SEM)	Time to peak amplitude (ms ± SEM)
Control	4.2 ± 0.8^a^	291 ± 78	9.1 ± 1.4^a^	147 ± 6
D1 agonist	5.9 ± 1.0^a^	355 ± 82	12.1 ± 2.5^a^	128 ± 12

The effects of the D1 agonist on maximum amplitude and time to peak amplitude at 10 Hz and 50 Hz are tabled.

a Significant difference between control and D1 receptor activation (*P* < 0.05; RM ANOVA).

**Table 3:** D1 receptor activation has no effect on layer I evoked non‐NMDA EPSPs

	10 Hz Stimulation		50 Hz Stimulation	
	Max amplitude from baseline (mV ± SEM)	Time to peak amplitude (msec ± SEM)	Max amplitude from baseline (mV ± SEM)	Time to peak amplitude (msec ± SEM)
Control	9.8 ± 0.8	175 ± 14	15.9 ± 1.4	74 ± 7
D1 agonist	10.1 ± 1.3	213 ± 42	14.5 ± 1.8	67 ± 8

The effects of the D1 agonist on maximum amplitude and time to peak amplitude at 10 Hz and 50 Hz are tabled.

**Table 4:** D1 receptor activation has frequency‐dependent effects on layer I evoked NMDA EPSPs

	10 Hz stimulation		50 Hz stimulation	
	Max amplitude from baseline (mV ± SEM)	Time to peak amplitude (msec ± SEM)	Max amplitude from baseline (mV ± SEM)	Time to peak amplitude (msec ± SEM)
Control	4.0 ± 0.6^a^	247 ± 48	5.9 ± 1.4	114 ± 7
D1 agonist	2.4 ± 0.6^a^	388 ± 64	6.7 ± 0.9	118 ± 11

The effects of the D1 agonist on maximum amplitude and time to peak amplitude at 10 Hz and 50 Hz are tabled.

a Significant difference between control and D1 receptor activation (*P* < 0.05; RM ANOVA).

**Table 5:** Summary of D1 receptor effects on layer V and layer I evoked NMDA and Non‐NMDA receptor‐mediated responses in Type I and Type II pyramidal cells

	Layer V stimulation															
	NMDA								Non‐NMDA							
	EPSP amplitude		EPSP ratio		Time to peak amplitude		Max amplitude		EPSP amplitude		EPSP Ratio		Time to peak amplitude		Max amplitude	
cell type	High frequency	Low frequency	High frequency	Low frequency	High frequency	Low frequency.	High frequency.	Low frequency	High frequency	Low frequency	High frequency	Low frequency	High frequency	Low frequency	High frequency	Low frequency
Type I Cells	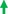	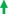	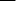	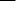	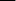	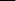	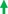	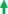	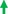	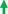	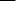	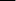	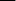	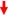	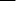	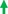
Type II Cells	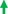	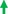	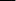	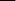	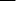	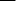	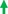	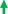	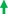	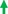	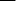	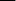	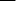	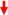	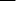	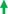

	Layer I stimulation															
	NMDA								Non‐NMDA							
	EPSP amplitude		EPSP ratio		Time to peak amplitude		Max amplitude		EPSP amplitude		EPSP ratio		Time to peak amplitude		Max amplitude	
Cell Type	High frequency	Low frequency	High frequency	Low frequency	High frequency	Low frequency	High frequency	Low frequency	High frequency	Low frequency	High frequency	Low frequency	High frequency	Low frequency	High frequency	Low frequency
Type I Cells	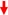	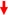	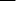	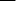	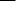	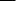	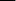	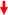	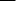	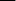	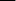	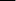	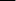	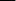	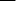	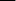
Type II Cells	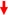	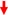	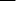	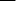	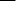	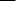	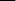	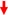	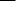	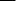	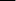	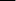	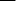	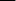	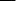	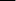

In contrast to non‐NMDAR‐mediated EPSPs, NMDAR‐mediated EPSPs evoked by layer V and layer I stimulation showed no difference in EPSP2/EPSP1 ratio between 10 Hz and 50 Hz. However, for NMDAR‐mediated EPSPs evoked by both layer V and layer I stimulation, the maximal amplitude reached during the train was higher, and the latency to peak was shorter, at 50 Hz compared to 10 Hz (Fig. 4C and D, Tables 1, 2, 3, 4, 5). Like non‐NMDAR‐mediated EPSPs, these results also indicate that, regardless of layer stimulation, temporal summation of NMDAR‐mediated EPSPs is more robust at higher frequencies.

## D1 receptor effects on EPSP trains

### Layer V evoked non‐NMDAR‐mediated EPSPs

**Figure 5:**
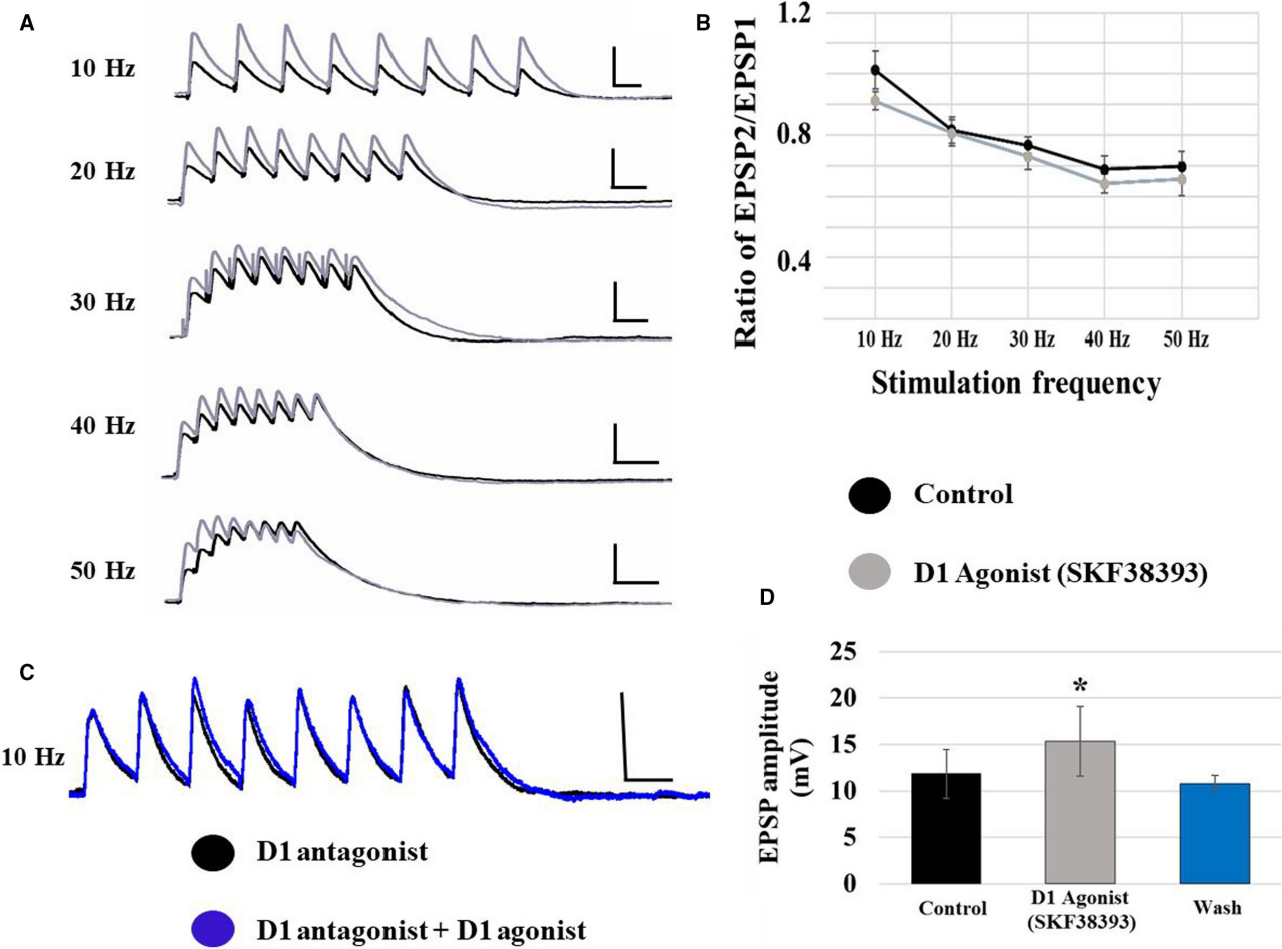
D1 receptor effects on non‐NMDA EPSPs evoked by layer V stimulation. (A) D1 receptor activation with SKF38393 significantly increases the amplitude of non‐NMDA EPSPs in all (both type I and type II) pyramidal neurons. Traces are shown for each stimulation frequency (10–50 Hz from top to bottom; control: black traces; SKF38393: gray traces; scale bars: *x* = 100 msec; *y* = 10 mV). (B) The EPSP amplitude ratio of EPSP2/EPSP1 was unaffected by the D1 agonist at any frequency. (C) The effects of SKF38393 on EPSP amplitude are blocked in the presence of the D1 antagonist, SCH23390. (D) The enhancement of EPSP amplitude caused by SKF38393 was completely reversed within 5‐min of drug washout.

The D1 agonist, SKF38393, increased the initial non‐NMDA EPSP amplitude in both cell types similarly; therefore, data are presented as an average of the two cell types. Initial EPSP amplitude was increased from 7.0 ± 0.8 mV to 15.3 ± 1.9 mV at 10 Hz (*P* < 0.05; *n* = 14). Representative non‐NMDA EPSP trains recorded from layer V stimulation are shown in control solution and in the presence of the D1 agonist (Fig. 5A, black and gray traces, respectively) for all frequencies. This increase in non‐NMDA initial EPSP amplitude by D1R activation was blocked by co‐application of the D1R antagonist, SCH23390 (at 10 Hz: D1 antagonist: 12.7 ± 1.1 mV; D1 agonist: 12.8 ± 1.7 mV; *P* > 0.05; *n* = 7; Fig. 5C). Additionally, a 5‐min activation of dopamine receptors may lead to changes that outlast the direct effects of downstream signaling events. Thus, we wanted to explore whether the effects of D1 receptor activation were reversible. Non‐NMDA EPSP trains were measured at 10 Hz in response to layer V stimulation in control solution, following a 5 min application of SKF38393 and a 5 min agonist washout. The enhancement of EPSP amplitude observed in the presence of SKF38393 was completely reversed within 5 min of drug washout (at 10 Hz: control: 11.8 ± 2.6 mV; D1 agonist: 15.3 ± 3.7 mV; wash: 10.8 ± 0.8 mV; *n* = 6; Fig. 5D).

The effects of D1 receptor activation on non‐NMDAR‐mediated short‐term synaptic dynamics were frequency‐dependent. SKF38393 application had no effect on the EPSP2/EPSP1 ratio at any frequency (Fig. 5B). However, D1 receptor activation significantly increased the maximum amplitude reached during the train and shortened the latency to peak amplitude at 10 Hz, but not at higher frequencies (data for 10 Hz and 50 Hz shown in Table 1; Superscripted ones indicate *P* < 0.05; RM ANOVA). These effects of D1 receptor activation on maximum amplitude reached were blocked by co‐application of the D1 antagonist (at 10 Hz, maximum amplitude: D1 antagonist: 12.7 ± 1.1 mV; D1 agonist: 12.8 ± 1.7 mV; *n* = 7). Additionally, these effects were reversed within 5 min of drug washout (at 10 Hz: control: 12.5 ± 1.4 mV; D1 agonist 15.3 ± 1.8 mV; wash: 9.6 ± 1.2 mV; *n* = 6). Furthermore, the effects of D1 receptor activation on latency to peak amplitude were also blocked by co‐application of the D1 antagonist (at 10 Hz, D1 antagonist: 142.9 ± 29.7 msec; D1 agonist: 228.6 ± 99.3 msec; *n* = 7) and were reversed within 5 min of drug washout (at 10 Hz: control: 116.7 ± 16.7 msec; D1 agonist 100 ± 0.0 msec; wash: 100 ± 0.0 msec).

### Layer V evoked NMDAR‐mediated EPSPs

**Figure 6:**
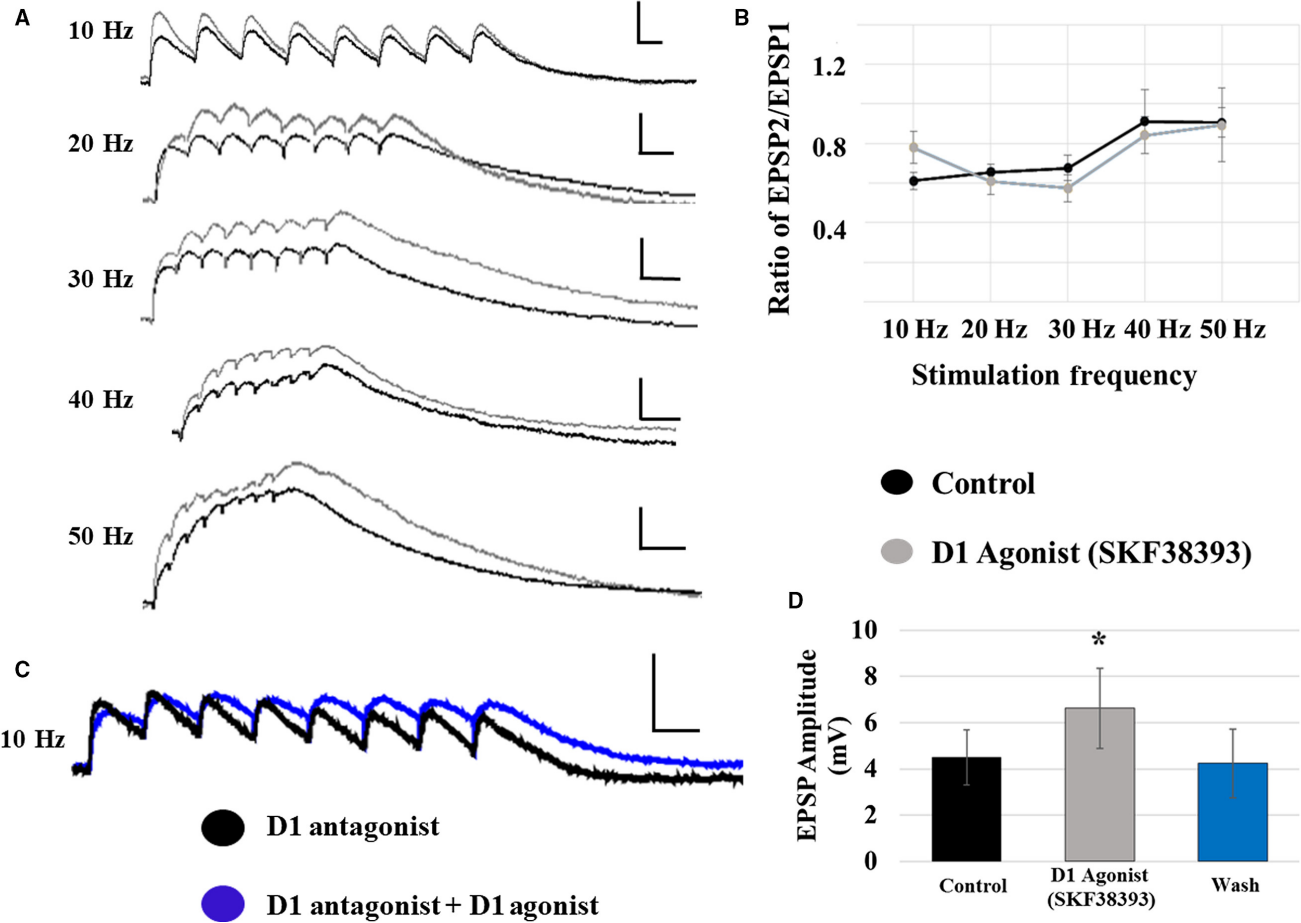
D1 receptor effects on NMDA EPSPs evoked by layer V stimulation. (A) D1 receptor activation with SKF38393 significantly increases the amplitude of NMDA EPSPs in all (both type I and type II) pyramidal cells. Traces are shown for each stimulation frequency (10–50 Hz from top to bottom; control: black traces; SKF38393: gray traces; scale bars: *x* = 100 msec; *y* = 5 mV). (B) The EPSP amplitude ratio of EPSP2/EPSP1 was unaffected by the D1 agonist at any frequency. (C) The effects of SKF38393 on EPSP amplitude are blocked in the presence of the D1 antagonist, SCH23390. (D) The enhancement of EPSP amplitude caused by SKF38393 was completely reversed within 5‐min of drug washout.

The D1 agonist, SKF38393, increased the initial NMDA EPSP amplitude in both cell types similarly; therefore, data is presented as an average of the two cell types. D1 receptor activation increased the initial NMDA EPSP amplitude from 4.0 ± 0.9 mV to 5.0 ± 1.0 mV at 10 Hz (*P* < 0.05; *n* = 20). Representative traces of NMDAR‐mediated EPSP trains recorded following layer V stimulation are shown in control solution and in the presence of the D1 agonist (Fig. 6A, black and gray traces, respectively). This increase in NMDA initial EPSP amplitude was blocked by co‐application of the D1R antagonist, SCH23390 (at 10 Hz, D1 antagonist: 4.5 ± 0.8 mV; D1 agonist: 4.3 ± 0.8 mV; *P* > 0.05; *n* = 7; Fig. 6C). Additionally, the enhancement of initial NMDA EPSP amplitude, observed in the presence of SKF38393, was completely reversed within 5 min of drug washout (at 10 Hz, control: 4.5 ± 1.2 mV; D1 agonist: 6.6 ± 1.7 mV; wash: 4.2 ± 1.5 mV; *n* = 7; Fig. 6D).

In contrast with non‐NMDA receptor‐mediated responses, the effects of D1 receptor activation on NMDAR‐mediated synaptic responses were not frequency‐dependent. SKF38393 application had no significant effect on the EPSP2/EPSP1 ratio at any frequency (Fig. 6B). However, D1 receptor activation increased the maximum amplitude reached during the train at all frequencies, yet had no effect on time to peak (data for 10 Hz and 50 Hz are shown in Table 2; Superscripted ones indicate *P* < 0.05; RM ANOVA; *n* = 20). These effects of D1 receptor activation were blocked by co‐application of the D1 antagonist (at 10 Hz, maximum amplitude: D1 antagonist: 4.5 ± 0.8 mV; D1 agonist: 4.3 ± 0.8 mV; *n* = 7). Additionally, these effects were reversed within 5 min of drug washout (at 10 Hz, maximum amplitude: control: 4.6 ± 1.2 mV; D1 agonist 6.8 ± 1.7 mV; wash: 4.6 ± 1.4 mV; *n* = 7).

### Layer I evoked non‐NMDAR‐mediated EPSPs

**Figure 7:**
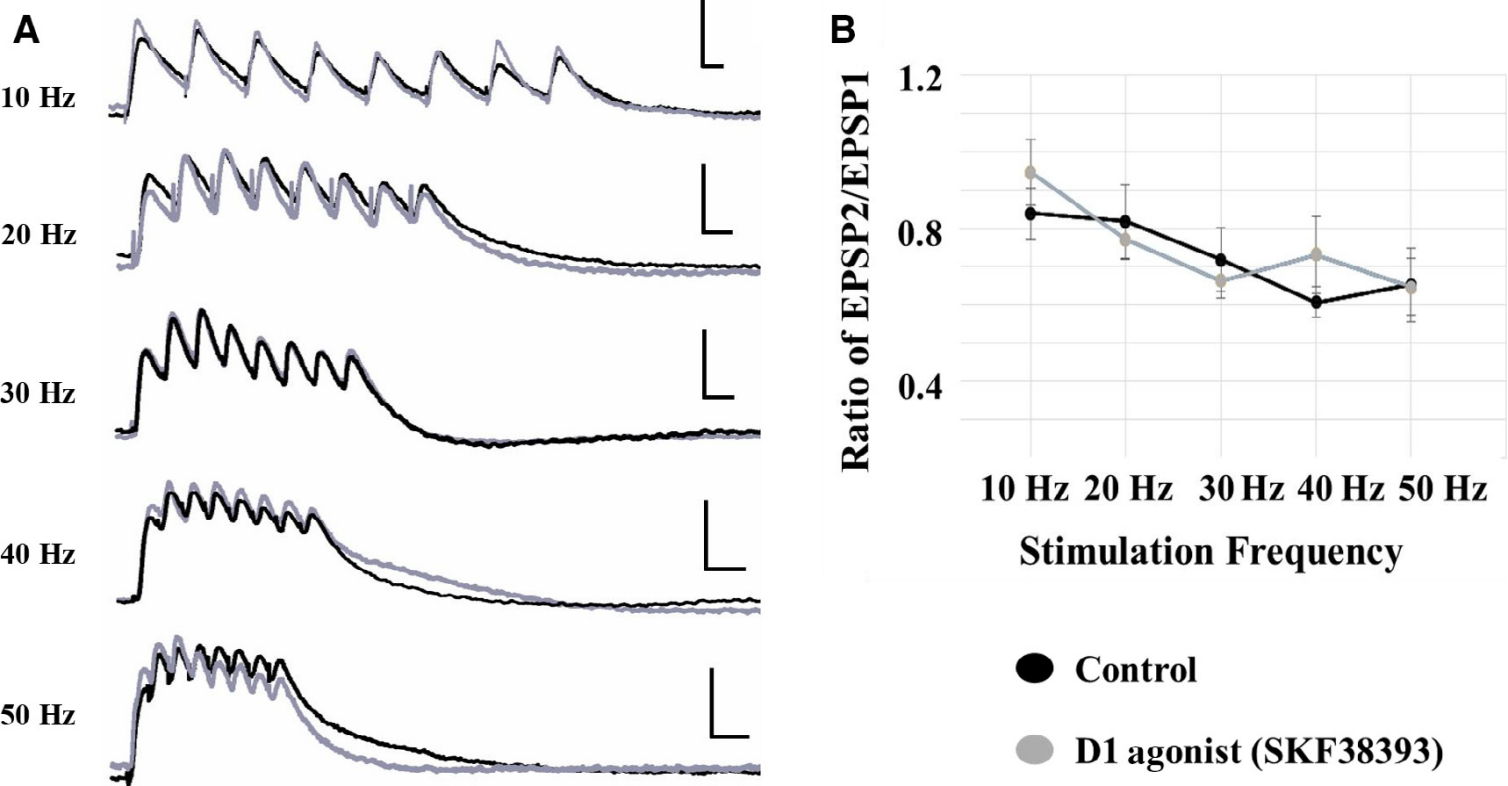
D1 receptor effects on non‐NMDA EPSPs evoked by layer I stimulation. (A) D1 receptor activation with SKF38393 had no effect on the amplitude of non‐NMDA EPSPs in either pyramidal cell type. Traces are shown for each stimulation frequency (10–50 Hz from top to bottom; control: black traces; SKF38393: gray traces; scale bars: *x* = 100 msec; *y* = 10 mV). (B) The EPSP amplitude ratio of EPSP2/EPSP1 was unaffected by the D1 agonist at any frequency.

D1 receptor activation had no significant effects on non‐NMDAR EPSP trains evoked by layer I stimulation in either cell type. Thus, data are presented as an overall average of the two cell types. The D1 agonist did not alter the initial EPSP amplitude (at 10 Hz, control: 4.0 ± 0.9 mV D1 agonist: 5.0 ± 1.0 mV (*P* > 0.05; *n* = 16)) or the ratio of EPSP2/EPSP at any frequency (Fig. 7A and B). Furthermore, SKF38393 had no effect on maximum amplitude reached during the train or latency to peak amplitude at any frequency compared with controls (data shown for 10 and 50 Hz; Table 3).

### Layer I evoked NMDAR‐mediated EPSPs

**Figure 8:**
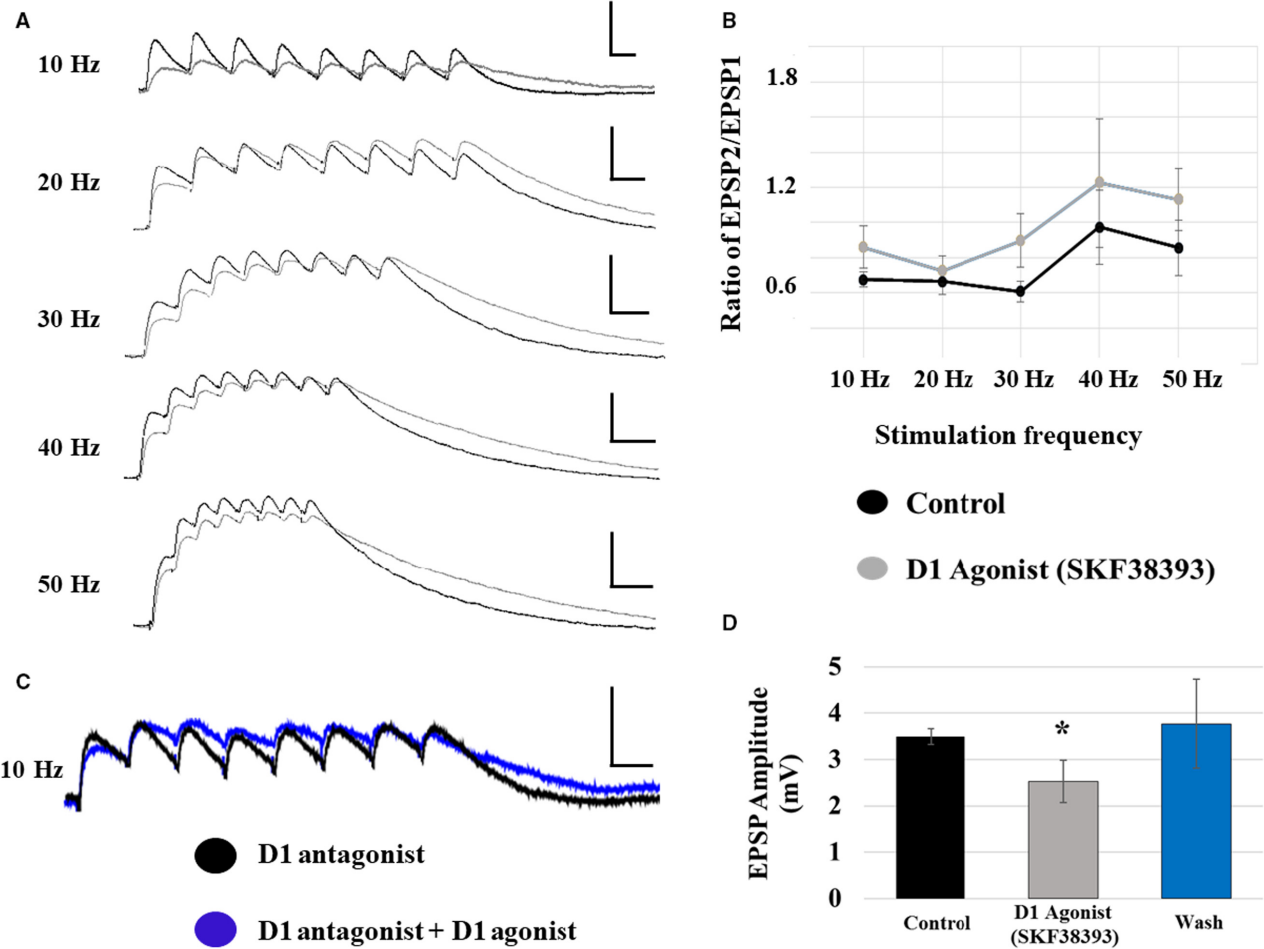
D1 receptor effects on NMDA EPSPs evoked by layer I stimulation. (A) D1 receptor activation with SKF38393 significantly decreased the amplitude of NMDA EPSPs in all (both type I and type II) pyramidal cells. Traces are shown for each stimulation frequency (10–50 Hz from top to bottom; control: black traces; SKF38393: gray traces; scale bars: *x* = 100 msec; *y* = 5 mV). (B) The EPSP amplitude ratio of EPSP2/EPSP1, in either cell type, was unaffected by the D1 agonist at any frequency. (C) The effects of SKF38393 on EPSP amplitude are blocked in the presence of the D1 antagonist, SCH23390. (D) The decrease in EPSP amplitude caused by SKF38393 was completely reversed within 5‐min of drug washout.

The D1 agonist, SKF38393, decreased the initial EPSP amplitude in both cell types similarly; therefore, data are presented as an average of the two cell types. Initial EPSP amplitude was decreased from 5.1 ± 1.0 mV to 1.5 ± 0.3 mV at 10 Hz (*P* < 0.05; *n* = 8). Representative NMDA EPSP traces recorded from layer I stimulation are shown in control solution and in the presence of the D1 agonist for all frequencies (Fig. 8A, black and gray traces, respectively). This decrease in initial EPSP amplitude was blocked by co‐application of the D1R antagonist, SCH23390 (at 10 Hz, D1 antagonist: 4.1 ± 0.6 mV; D1 agonist: 3.5 ± 0.5 mV; *P* > 0.05; *n* = 7; Fig. 8C). Additionally, the decrease in initial EPSP amplitude observed in the presence of SKF38393 was completely reversed within 5 min of drug washout (at 10 Hz, control: 3.5 ± 0.2 mV; D1 agonist: 2.5 ± 0.5 mV; wash: 3.7 ± 1.0 mV; *P* > 0.05; RM ANOVA; *n* = 4; Fig. 8D).

The effects of D1 receptor activation on NMDAR‐mediated short‐term synaptic responses were frequency‐dependent. SKF38393 application had no effect on the EPSP2/EPSP1 ratio at any frequency (Fig. 8B). However, D1 receptor activation decreased the maximum train amplitude at 10 Hz, but not at higher frequencies and had no effect on latency to peak amplitude at any frequency (data for 10 Hz and 50 Hz are shown in Table 4; Superscripted ones indicate *P* < 0.05; RM ANOVA). These effects of D1 receptor activation were blocked by co‐application of the D1 antagonist (at 10 Hz, maximum amplitude: D1 antagonist: 4.1 ± 0.6 mV; D1 agonist: 3.5 ± 0.5 mV; *n* = 7). Additionally, these effects were reversed within 5 min of drug washout (at 10 Hz: control: 3.5 ± 0.2 mV; D1 agonist 2.5 ± 0.5 mV; wash: 5.6 ± 0.9 mV; *n* = 4).

## Discussion

### Summary

In this study, type I (or PT) and type II (or IT) layer V pyramidal neurons were distinguished based on their electrophysiological properties and the effects of D1 receptor activation on high‐frequency synaptic trains evoked by apical tuft (layer I) or basal dendritic (layer V) stimulation were characterized. The main results were as follows: (1) Non‐NMDAR‐ and NMDAR‐mediated EPSPs differ in frequency‐dependent properties; non‐NMDAR‐mediated EPSPs are more depressing at high frequencies compared with low, whereas NMDAR‐mediated EPSPs show similar depression at all frequencies. (2) With layer V stimulation, D1 receptor activation increased both the non‐NMDAR‐ and NMDAR‐mediated initial EPSP amplitude in both type I and type II cells. The peak amplitude of the train was also enhanced for both non‐NMDA and NMDAR‐mediated EPSPs and the time to reach peak amplitude was decreased in non‐NMDAR‐mediated EPSPs with D1 receptor activation at low, but not high frequencies. (3) D1 receptor activation had no significant effect on layer I‐evoked non‐NMDAR‐mediated EPSP amplitude, peak train amplitude or time to peak at any frequency. (4) D1 receptor activation decreased the initial amplitude of layer I evoked NMDAR‐mediated EPSPs, but had no effect on peak train amplitude or time to peak.

### Comparisons with previous studies

It is of interest that we did not observe any significant differences in synaptic dynamics in comparing identified type I and type II layer V pyramidal cells. Several studies have characterized short‐term EPSP dynamics at connections between layer V pyramidal cell subtypes; both facilitating and depressing patterns have been observed (Wang et al. 2006; Morishima et al. 2011; Lee et al. 2014). For example, Lee et al. (2014) found that commissural (ie. type II) inputs onto type I pyramidal cells exhibit facilitating EPSPs, but commissural inputs onto type II cells show depressing EPSPs. In contrast, Morishima et al. (2011) and Wang et al. (2006), using dual cell recordings, demonstrated that both type I and type II pyramidal cells can show facilitating and depressing EPSP patterns. In the present study, our stimulation protocol resulted in activation of a heterogeneous population of fibers, including other Layer V cells (both type I and II), axons from other cortical layers, and axons from other brain regions. Thus, one would expect a mixture of both facilitating and depressing EPSP dynamics at layer V synapses, and this may have obscured any differences that may exist between type I and II pyramidal cells. While the dual recording studies focused on synapses targeting primarily basal dendrites in layer V, the emphasis in our study was to characterize the dynamics at layer V synapses as compared with the dynamics at layer I synapses. To our knowledge, the short‐term dynamics of EPSPs evoked by layer I stimulation have not been characterized in the literature. However, Hempel et al. (2000) reported a short‐term decrease in amplitude of EPSC trains (i.e., depressing responses) recorded in layer V cells evoked by layer II/III stimulation.

The modulation of evoked EPSP short‐term dynamics by dopamine receptor activation has been studied to some extent (Law‐Tho et al. 1994; Yang 2000; Gao et al. 2001; Seamans et al. 2001; Young and Yang 2005; Matsuda et al. 2006; Rotaru et al. 2007; Xu and Yao 2010). However, these studies of dopamine effects on the amplitude of low‐frequency evoked EPSCs and EPSPs have often yielded diverse, and sometimes contradicting results. Consistent with our results, two studies have reported that dopamine produces short‐term potentiation of EPSP amplitude in layer V pyramidal cells (Matsuda et al. 2006; Xu and Yao 2010). Additionally, D1 receptor activation has also been shown to increase EPSC amplitude (Yang 2000; Seamans et al. 2001) and EPSP amplitude (Rotaru et al. 2007) at layer V pyramidal synapses evoked by layer V stimulation. Seamans et al. (2001) also reported that D1 receptor activation enhances both isolated NMDAR and non‐NMDAR‐mediated EPSC components in layer V pyramidal cells of rat mPFC evoked by layer V stimulation, similar to our current findings.

Although many studies have shown similar results to our study, others have reported contradicting findings. For example, studies have shown that D1 receptor activation depressed EPSP amplitude in layer V pyramidal neurons evoked by layer V (Gao et al. 2001) and layer VI (Law‐Tho et al. 1994) stimulation. Furthermore, Law‐Tho et al. (1994) have reported a similar effect on isolated EPSP components in rat mPFC layer V pyramidal cells evoked by layer VI stimulation. Lastly, others have reported D1 receptor activation to have no effect on layer III evoked EPSC amplitude in layer V pyramidal neurons (Young and Yang 2005). To our knowledge, the present study is the first to characterize the effects of D1 receptor activation on the frequency‐dependent properties of *isolated* NMDA and non‐NMDA EPSP components, evoked by *layer I versus layer V* evoked stimulation.

### Spatial distribution of dopamine receptors and potential subcellular mechanisms

Dopaminergic receptor expression is known to vary throughout the cortical layers of the prefrontal cortex. It has been shown that both D1 and D2 receptors show higher expression levels within deeper layers, specifically layers V and VI (Bergson et al. 1995; Santana et al. 2009). Additionally, it has been shown that D1 and D2 receptor expression levels are similar between receptor subtypes in deep layers, but expression levels of D2 receptors was higher compared with D1 receptors within superficial layer I (Vincent et al. 1993). Interestingly, others have shown that D1 receptor expression within layer I is extensive on glial cells, a phenomenon not observed within layer V (Dombrowski et al. 2001). Despite these differences between layer I and layer V in regards to distribution of dopaminergic receptors, it is currently unknown whether dopamine acts primarily through presynaptic or postsynaptic receptors to affect changes in non‐NMDA‐ or NMDA receptor‐mediated currents. While our current study was not designed to directly address subcellular mechanisms of action, here we summarize results from previous studies that may lend some insight into the mechanisms of D1 receptor actions on glutamatergic synapses observed in this study.

First, several studies have suggested that D1 receptor activation increases AMPA receptor trafficking into the postsynaptic membrane, giving rise to an increase in AMPA EPSP amplitude (Chao et al. 2002; Sun et al. 2005; Gao et al. 2006) as observed at layer V synapses in the current study. Other studies have suggested that D1 receptor activation may enhance NMDA receptor function through a cAMP/PKA‐dependent regulation of phosphorylation states (Schoffelmeer et al. 2000), which could account for the increase in NMDA EPSP amplitude with layer V stimulation. Thus, D1 receptor‐mediated modulation of long‐term potentiation may involve an increase in NMDA receptor function mediated through the cAMP/PKA pathway. Similarly, other studies have suggested that D1R activation, acting through the PKA pathway, modulates persistent sodium currents, leading to an increase in soma excitability (Gorelova and Yang 2000). Similarly Rotaru et al. (2007) also suggested that D1 receptor activation has an enhancing effect on the slowly activating persistent sodium current, while simultaneously attenuating the slowly activating potassium current. In combination, this study showed that these D1 receptor‐induced alterations can lead to an enhancement of local connections (Yang and Seamans 1996), an effect observed at layer V synapses in the current study. The higher expression levels of D1 receptors in deep layers could account for the fact that we saw no significant effect of D1 receptor activation on non‐NMDA receptor‐mediated responses in layer I; however, this cannot account for why initial NMDA receptor‐mediated EPSPs were attenuated. We address below mechanisms that may account for these observations.

D1 receptor activation has also been shown to have regional effects within the prefrontal cortex. D1 receptor activation has been shown to attenuate high‐threshold calcium currents (Seamans et al. 1997; Zahrt et al. 1997), which are known to amplify signals that propagate along the dendrite to reach the soma (Kim and Connors 1993; Yuste et al. 1994; Seamans et al. 1997). Furthermore, high‐threshold calcium channels allow the apical segment to act as a current amplifier for distal synaptic events as well as a dendritic trigger zone (Yuste et al. 1994), a phenomenon inhibited by D1 receptor activation. These findings suggest that, through attenuation of apical dendritic high‐threshold calcium currents, D1 receptor activation restricts the inputs of apical dendrites on layer V pyramidal neurons. These previous studies (Seamans et al. 1997; Zahrt et al. 1997) and our results support the hypothesis that D1 receptor activation leads to intrinsic changes in layer V pyramidal neurons, increasing the strength of layer V inputs, and inhibiting inputs from layer I. Importantly, in the current study we compared the effects of D1 receptor activation on both superficial and deep layer synaptic responses in layer V pyramidal cells, providing stronger support for this hypothesis.

At layer V synapses, we observed that D1 receptor activation had differential effects on non‐NMDA receptor‐mediated synaptic responses depending on train frequency. D1 receptor activation increased the maximum amplitude of summated non‐NMDA receptor‐mediated EPSPs at low frequency (10 Hz) but not at higher frequency (50 Hz). Since D1 receptor activation increased the initial EPSP amplitude, we believe that the simplest explanation for the frequency effect is that transmitter release was enhanced by D1 receptor activation, leading to more rapid depletion of release at higher train frequencies. This does not preclude any postsynaptic effects that D1 receptor activation may have, as discussed above; rather, that presynaptic changes may also occur with D1 receptor activation (for review, see Seamans and Yang (2004)).

Regarding NMDA receptor‐mediated EPSPs, D1 receptor activation had opposing effects on layer I versus layer V synaptic responses. At layer I synapses, where D1 receptor activation decreased the initial EPSP amplitude, the maximum amplitude of summated NMDA receptor‐mediated EPSPs was decreased at low frequency but not at high frequency during D1 receptor activation. The longer time course of NMDA receptor‐mediated EPSPs may account for greater summation at high train frequencies, in spite of the initial decrease in EPSP amplitude. As discussed above, since D1 receptor activation likely inhibits active currents near the apical tufts, it is also possible that these effects are frequency‐dependent, inhibiting train summation more at low frequencies. In contrast, at layer V synapses, D1 receptor activation enhanced initial EPSP amplitude and increased the maximum amplitude of summated EPSPs at all train frequencies. If D1 receptor activation increases the probability of transmitter release at layer V synapses, depletion of release at higher frequencies may counteract a greater summation of the longer time course NMDA receptor‐mediated EPSPs at high train frequency.

Overall, D1 receptor activation appears to enhance synaptic responses at layer V synapses, while attenuating responses in the tufts. The precise locations of these changes, however, remain to be determined by more rigorous studies designed to interrogate mechanisms of presynaptic and postsynaptic alterations, and are likely complex.

### Implications for prefrontal cortical function

The mPFC is highly interconnected with other neocortical and subcortical regions. This complex interconnectivity may play a role in long‐range reverberant activity, initiating and maintaining the persistent activity required for many prefrontal executive functions. Additionally, it has been suggested that both intrinsic and extrinsic factors are likely important for generating persistent neuronal activity within the prefrontal cortex and may be heavily reliant on dopaminergic modulation (Scheler and Fellous 2001; Wang and O'Donnell 2001; Durstewitz and Seamans 2002), with proper levels of dopamine essential for PFC memory‐related tasks. However, the mechanistic details underlying this phenomenon are incompletely understood. The current study suggests that dopamine, through D1 receptor activation, has compartmentalized effects on non‐NMDAR‐ and NMDAR‐mediated EPSPs on layer V pyramidal neurons. Our results suggest that D1 receptor activation increases the influence of local inputs from other layer V pyramids, while restricting the influence of layer I (tuft) inputs on spike firing at the soma. It has also been suggested by others that D1 receptor facilitation of the NMDA EPSP component (as observed in our study) may favor recurrent connections between pyramidal neurons in close proximity (Rotaru et al. 2007). Additionally, our results suggest that D1 receptor activation increases the ability of a cell to summate in response to layer V stimulation (primarily recurrent connections between pyramidal cells) at low frequencies (i.e., 10 Hz). This summation may enhance the ability of layer V cells to integrate information that contributes to persistent firing, for example by facilitating NMDA “spikes” in the basal dendrites (Larkum et al. 2009). Our results are also consistent with the hypothesis proposed by Yang and Seamans (Yang and Seamans 1996) that D1 receptor activation in layer V pyramidal cells suppresses synaptic input to the apical tufts in layer I while augmenting synaptic inputs to basal dendrites by altering intrinsic ion currents. We further hypothesize that dopamine, acting via D1 receptors, may play a role in strengthening selected local connections within layer V, enhancing output from a specific ensemble via NMDAR‐dependent plasticity, while inhibiting plasticity at layer I synapses. One role of D1 receptor activation in modulating memory functions may thus be to facilitate choice selection by layer V output neurons, while inhibiting or stabilizing “top‐down” influences impinging on layer I.

## Conclusion

Our results are consistent with the hypothesis that dopamine, through activation of D1 receptors on layer V cells in mPFC, may play a role in stabilizing top‐down (contextual) information while promoting local (bottom‐up) influences and plasticity during memory‐related tasks. Thus, information impinging onto the basal dendrites is processed differently than information targeting the apical tufts of pyramidal cells. Additionally, results from the current study and our previous study (Leyrer‐Jackson and Thomas 2017), characterizing the effects of D2 receptor activation on short‐term, frequency‐dependent synaptic dynamics, lend support to the hypothesis that dopamine has *compartment specific* effects on layer V pyramidal neurons that are dependent on differential D1 and D2 receptor activation. In contrast to D1 receptor activation, D2 receptor activation had no effect on local layer V activity, but may lead to amplification of salient contextual information arriving in the apical tufts by inhibiting low‐frequency inputs and facilitating high‐frequency inputs (Leyrer‐Jackson and Thomas 2017). Taken together, these results suggest that with optimal dopamine levels, in the basal dendritic compartment, persistent activity and synaptic plasticity may be promoted for specific environmental information relevant to a memory task, while in the apical tuft compartment, salient contextual information is amplified appropriately.

## Conflict of Interest

The authors declare that the research was conducted in the absence of any commercial or financial relationships that could be construed as a potential conflict of interest.
